# Anti-fibrotic effects of nintedanib in lung fibroblasts derived from patients with idiopathic pulmonary fibrosis

**DOI:** 10.1186/s12931-014-0157-3

**Published:** 2014-12-12

**Authors:** Katrin E Hostettler, Jun Zhong, Eleni Papakonstantinou, George Karakiulakis, Michael Tamm, Petra Seidel, Qingzhu Sun, Jyotshna Mandal, Didier Lardinois, Christopher Lambers, Michael Roth

**Affiliations:** Pulmonary Cell Research, Department of Biomedicine, University Hospital Basel, Basel, 4031 Switzerland; Clinics of Respiratory Medicine, University Hospital Basel, Petersgraben 4, Basel, 4031 Switzerland; Department of Pharmacology, School of Medicine, Aristotle University of Thessaloniki, Thessaloniki, 54124 Greece; Clinics of Thoracic Surgery, University Hospital Basel, Basel, 4031 Switzerland; Department of Internal Medicine IV, University of Vienna, Vienna, 1090 Austria

**Keywords:** In vitro model, Kinase inhibitor, Lung fibrosis, Fibroblasts

## Abstract

**Background:**

Idiopathic pulmonary fibrosis (IPF) is a progressive lung disease with poor prognosis. The kinase inhibitor nintedanib specific for vascular endothelial growth factor receptor (VEGFR), platelet-derived growth factor receptor (PDGFR) and fibroblast growth factor receptor (FGFR) significantly reduced the rate of decline of forced vital capacity versus placebo.

**Aim:**

To determine the in vitro effect of nintedanib on primary human lung fibroblasts. Methods: Fibroblasts were isolated from lungs of IPF patients and from non-fibrotic controls. We assessed the effect of VEGF, PDGF-BB and basic FGF (bFGF) ± nintedanib on: (i) expression/activation of VEGFR, PDGFR, and FGFR, (ii) cell proliferation, secretion of (iii) matrix metalloproteinases (MMP), (iv) tissue inhibitor of metalloproteinase (TIMP), and (v) collagen.

**Results:**

IPF fibroblasts expressed higher levels of PDGFR and FGFR than controls. PDGF-BB, bFGF, and VEGF caused a pro-proliferative effect which was prevented by nintedanib. Nintedanib enhanced the expression of pro-MMP-2, and inhibited the expression of TIMP-2. Transforming growth factor-beta-induced secretion of collagens was inhibited by nintedanib.

**Conclusion:**

Our data demonstrate a significant anti-fibrotic effect of nintedanib in IPF fibroblasts. This effect consists of the drug’s anti-proliferative capacity, and on its effect on the extracellular matrix, the degradation of which seems to be enhanced.

## Introduction

Idiopathic pulmonary fibrosis (IPF) is the most common form of idiopathic interstitial pneumonias, and it is characterized by a progressive decline in lung function, poor survival and limited therapeutic options. The pathogenesis remains incompletely understood, but aberrant wound healing, resulting in progressive lung injury and scarring seem to play a pivotal role [1].

It is indicated that fibroblasts play a central role in the pathogenesis of fibrotic processes, and several factors influence their proliferation and extracellular matrix (ECM) synthesis [1]. In IPF, these mesenchymal cells have an increased activity and response to fibrogenic cytokines [1]. Several growth factors are suggested to be pivotal in the development of IPF, including platelet-derived growth factor (PDGF), basic fibroblast growth factor (bFGF), and vascular endothelial growth factor (VEGF). PDGF is a fibrogenic mediator [2], and was increased in a murine IPF-animal model [3]. The inhibition of PDGF signalling reduced bleomycin-induced pulmonary fibrosis in mice [4]. bFGF is a potent mitogenic factor and high levels of bFGF were found in lung tissue derived from patients with IPF [5]. And finally, blocking VEGF signalling reduced lung fibrosis in a mouse model [6].

Nintedanib (BIBF 1120) is an orally available indolinone derivate that competitively binds to the ATP-binding sites within the kinase domains of VEGF receptor (VEGFR)1, VEGFR2, VEGFR3, FGF receptor (FGFR)1, FGFR3, and PDGF receptor (PDGFR)α and PDGFRβ [7]. In vitro data demonstrated direct growth inhibitory activity of nintedanib in different cell lines [7].

Two randomized, placebo controlled, phase 3 trials (INPULSIS-1 and INPULSIS-2) evaluating the efficacy and safety of nintedanib in patients with IPF demonstrated that nintedanib-treatment reduced the decline in forced vital capacity, which conforms to a slow-down of disease progression [8]. Furthermore, in one trial (INPULSIS-2), there was a significant benefit with nintedanib versus placebo with regard to the time to the first acute exacerbation [8].

So far, the effect of nintedanib on primary human lung cells derived from patients with IPF has not been explored. In the present study, we determined the in vitro effect of nintedanib on the proliferative capacity and collagen synthesis by primary human lung fibroblasts derived from IPF lungs and non-fibrotic controls.

## Materials and methods

Nintedanib (BIBF 1120) ((methyl (3Z)-3-[({4-[N-methyl-2-(4-methylpiperazin-1-yl)acetamido]phenyl}amino)(phenyl) methylidene]-2-oxo-2,3-dihydro-1H-indole-6-carboxylate ethane sulfonate salt, batch # 1051764) was provided by Boehringer Ingelheim Pharma GmbH & Co. KG, Biberach, Germany.

### Patients and cell culture

Human lung tissue was obtained with the approval of the Human Ethics Committee of the University of Basel (Ref. Nr. EK:05/06), Switzerland, and with the written informed consent of each patient. Human primary lung cells were isolated, as reported previously [9], from lung biopsies of 4 patients diagnosed with IPF according to the recently published guidelines [10]. Non-fibrotic control cells were isolated from the macroscopically normal part of the lung of 4 patients undergoing therapeutic lung resection for carcinoma. All experiments were performed using cells at passage 3 to 6.

### Western blotting

IPF fibroblasts and non-fibrotic control cells were grown to 80% confluence and were then serum-starved for 24 hours. Cells were pre-incubated for 30 minutes with nintedanib (400 nM) before different stimuli (recombinant human PDGF-BB [10 ng/ml], recombinant human bFGF [10 ng/ml], recombinant human VEGF [10 ng/ml]: R&D Systems; Abingdon, United Kingdom) were added for another 30 minutes. Western blotting was performed as previously described [11]. Primary antibodies used: PDGFRα, VEGFR1, FGFR1, c-Abelson (c-Abl), extracellular signal-regulated kinase (ERK) 1/2, phosphorylated (pho) PDGFRα/β, pho-VEGFR2, pho-c-Abl, pho-ERK1/2 (Cell Signaling Technology, BioConcept; Allschwil, Switzerland) and pho-FGFR1α (Abcam; Cambridge, United Kingdom).

### Cell proliferation

Cells were seeded (10^4^ cells/ml) in 24-well plates and allowed to attach overnight before being serum starved (0.1% FCS, 24 hours). Cells were then exposed to different stimuli (recombinant human PDGF-BB [R&D Systems]; recombinant human bFGF [R&D Systems]; recombinant human VEGF [R&D Systems]) in the presence and absence of nintedanib (0.001, 0.01, 0.1, 1 μM) for 48 hours before being manually counted (Neubauer hematocytometer).

### Gelatin zymography

Confluent serum-deprived fibroblasts were stimulated with increasing concentrations of nintedanib (0.001, 0.01, 0.1, 1 μM) under standard conditions for 24 hours, and conditioned medium samples were collected. The gelatinolytic activity of matrix metalloproteinase (MMP)-2 and −9 was determined as described earlier [12].

### Enzyme-linked immunosorbent assay

Concentrations of MMP-2 and tissue inhibitor of metalloproteinase (TIMP)-2 were quantified in the conditioned medium using an enzyme-linked immunosorbent assay (ELISA) kit (R&D Systems). Sensitivity of the method employed was: MMP − 2: 0.16 ng/ml and TIMP-2: 0.011 ng/ml.

### ECM-deposition

Confluent serum-deprived fibroblasts were stimulated with 5 ng/ml human recombinant transforming growth factor-β (TGF-β) 1 (R&D Systems) and incubated in the presence or absence of nintedanib (0.001, 0.01, 0.1, 1 μM). The cells were further kept under standard conditions (37°C, 95% humidity, 5% CO_2_) for 48 hours. Collagen secretion and deposition (collagens type I to V) were determined using Sircol™ Assay kit (Biocolor; Carrickfergus, United Kingdom).

### Statistical analysis

Statistical comparisons were made by Student’s t-test. p-values ≤ 0.05 were considered significant. Where applicable, data are shown as mean ± standard error of the mean (SEM) from at least three independent experiments performed in duplicates or triplicates. Data representing image analysis are shown as representative immunoblots and as means SEM after densitometric image analysis.

## Results

### The effect of nintedanib on receptor expression and phosphorylation

**Figure 1:**
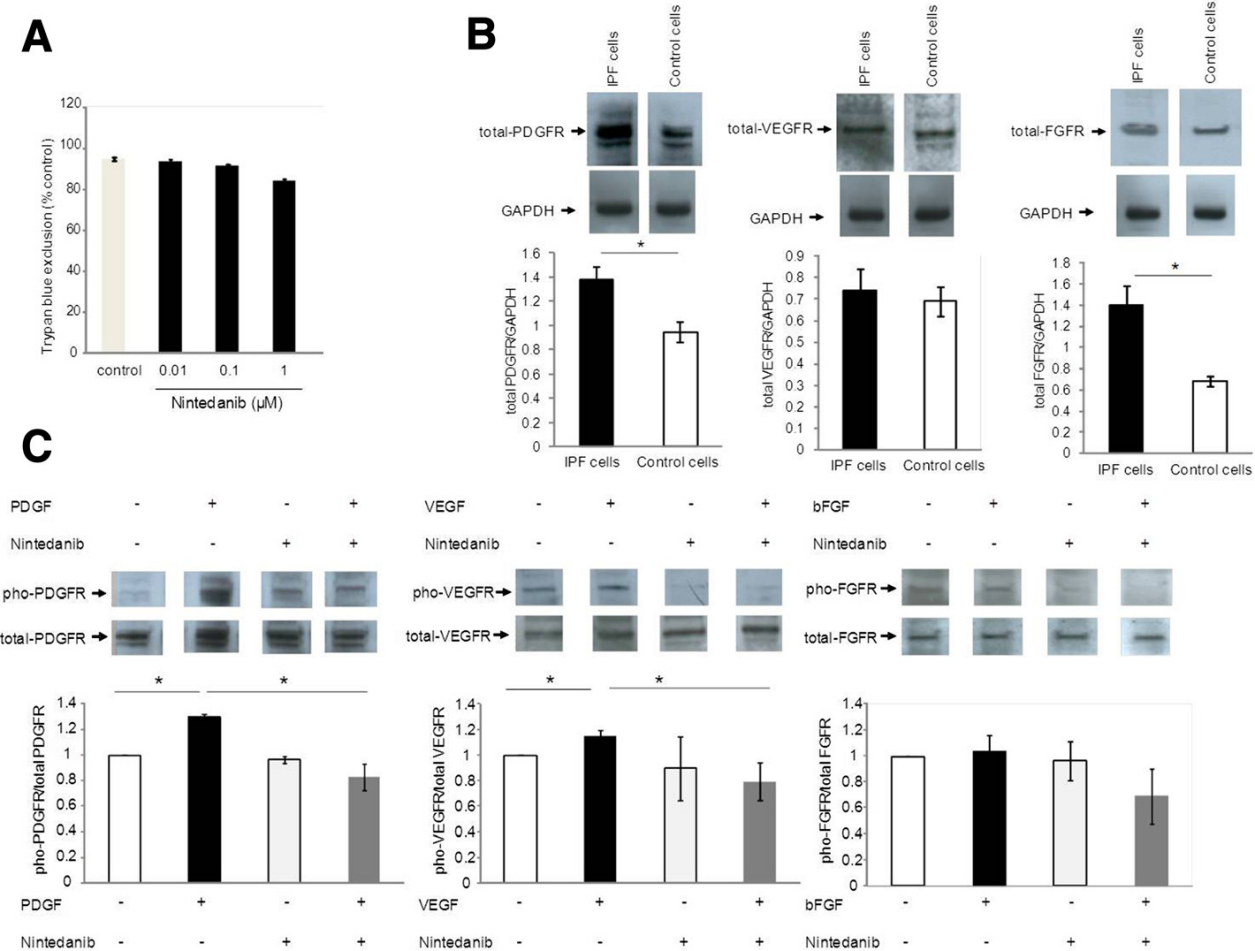
**Effect of nintedanib on receptor expression and phosphorylation. (A)** Fibroblasts derived from patients with IPF were treated with increasing concentrations of nintedanib (0.01, 0.1, and 1 μM) for 24 hours and cell viability was analysed by trypan blue exclusion staining. **(B)** Representative immuno-blots showing the expression of total PDGF-receptor (PDGFR), VEGF-receptor (VEGFR) and FGF-receptor (FGFR), in primary human lung IPF fibroblasts and non-fibrotic control cells. The bar charts summarise the densitometric analysis of total receptor expression normalised to GAPDH in IPF and control cells. Data are presented as mean ± SEM of experiment performed in three different cell lines. *p < 0.05. **(C)** Representative immuno-blots showing total and phosphorylated PDGFR, VEGFR, and FGFR in primary human lung IPF fibroblasts. Fibroblasts were starved for 24 hours. Before stimulation with PDGF-BB, bFGF, or VEGF (all 10 ng/ml) for 30 minutes, cells were pre-incubated with nintedanib (400 nM) for 30 minutes. The bar charts summarise the densitometric analysis of receptor phosphorylation normalised to total receptor expression in IPF cells. Data are presented as mean ± SEM of experiment performed in three different cell lines. *p < 0.05.

The viability of fibroblasts was not affected by the treatment with nintedanib at concentrations up to 1 μM as assessed by trypan blue exclusion staining (Figure 1A). There was no difference comparing the effect of nintedanib on viability in IPF cells versus non-fibrotic control cells (data not shown). IPF fibroblasts expressed higher levels of PDGFR and FGFR, as compared to non-fibrotic control cells (Figure 1B), whereas VEGFR was expressed at similar levels in fibrotic and non-fibrotic cells (Figure 1B). A significant inhibitory effect of nintedanib on phosphorylation of growth factor receptors by their specific ligands was observed for the PDGFR and the VEGFR in IPF cells (Figure 1C). High levels of phosphorylated FGFR were found in unstimulated cells which could not be further enhanced by stimulation with bFGF, but a down-regulation was detected in the presence of nintedanib in IPF cells (Figure 1C).

### Nintedanib inhibits growth-factor-induced proliferation of primary human lung fibroblasts

**Figure 2:**
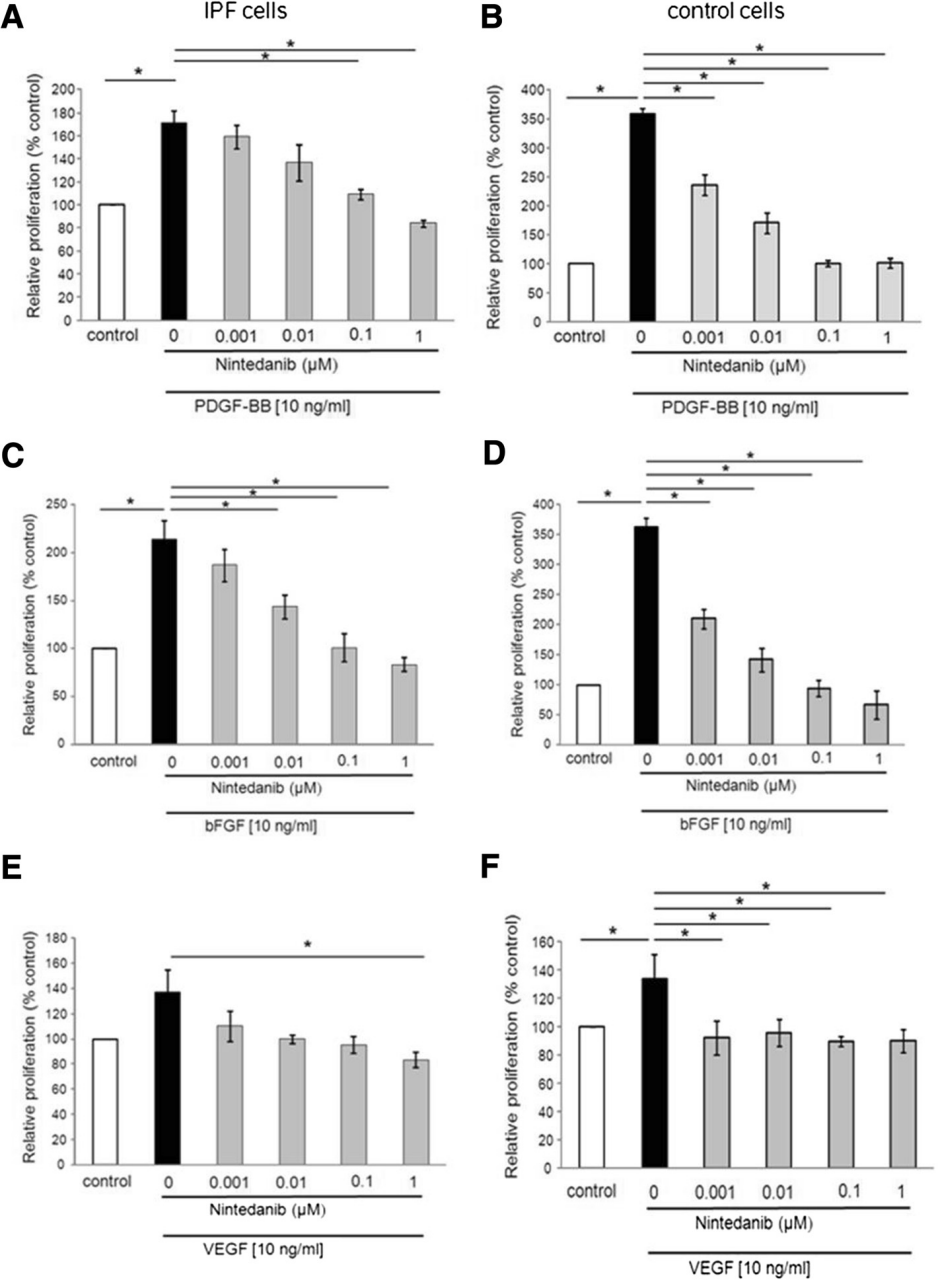
**Effect of nintedanib on PDGF-BB- (A, B), bFGF- (C, D), and VEGF- (E, F) induced fibroblast proliferation compared with serum-free RPMI (control) in fibroblasts derived from IPF lungs (A, C, E) and non-fibrotic control lungs (B, D, F).** Primary human lung fibroblasts derived from IPF patients and from non-fibrotic lungs were pre-incubated for 30 minutes with increasing concentrations of nintedanib (0.001, 0.01, 0.1, and 1 μM), before adding PDGF-BB (10 ng/ml), bFGF (10 ng/ml), and VEGF (10 ng/ml) for 48 hours. Serum-free RPMI served as control. Cell growth was assessed by manual cell counting, and values are presented as mean ± SEM of triplicate independent experiments performed in 4 different cell lines, expressed as relative proliferation compared to control (serum-free RPMI medium) which was set to 100%. *p < 0.05.

We observed a significant pro-proliferative effect of PDGF-BB (p < 0.05, Figure 2A,) and bFGF (p < 0.05, Figure 2C,) in primary IPF lung fibroblasts, as well as in cells from non-fibrotic control lungs (p < 0.05, Figure 2B [PDGF-BB]; p < 0.05, Figure 2D [bFGF]). The pro-proliferative effect induced by the two growth factors was significantly more pronounced in non-fibrotic control cells compared to IPF fibroblasts. VEGF also caused a significant mitogenic effect in non-fibrotic cells (p < 0.05, Figure 2F), but not in IPF-derived fibroblasts (Figure 2E). In IPF fibroblasts, the mitogenic effect induced by PDGF-BB (10 ng/ml) was significantly prevented by pre-incubation (30 minutes) with nintedanib, at concentrations of 0.1 μM (p < 0.05; Figure 2A) and 1 μM (p < 0.05; Figure 2A). In non-fibrotic cells, even lower concentrations of nintedanib (0.001 μM, 0.01 μM) significantly antagonized the PDGF-BB-induced pro-proliferative effect (p < 0.05; Figure 2B). Similar results were obtained in bFGF-stimulated fibroblasts. As shown in Figure 2C, nintedanib significantly prevented the bFGF-induced pro-proliferative effect in IPF fibroblasts at concentrations of 0.01, 0.1, and 1 μM (p < 0.05; Figure 2C). In non-fibrotic fibroblasts, even the lowest concentration of nintedanib (0.001 μM) prevented the bFGF-induced proliferation (p < 0.05; Figure 2D). In IPF fibroblasts, VEGF-induced proliferation was very low, as compared to PDGF-BB or bFGF-induced effects. However, it was significantly prevented by nintedanib at the highest concentration of 1 μM (p < 0.05; Figure 2E). Even more in non-fibrotic fibroblasts nintedanib completely prevented VEGF-induced proliferation already at a very low concentration of 0.001 μM (p < 0.05; Figure 2F).

### Effect of nintedanib on matrix metalloproteinases and their inhibitors

**Figure 3:**
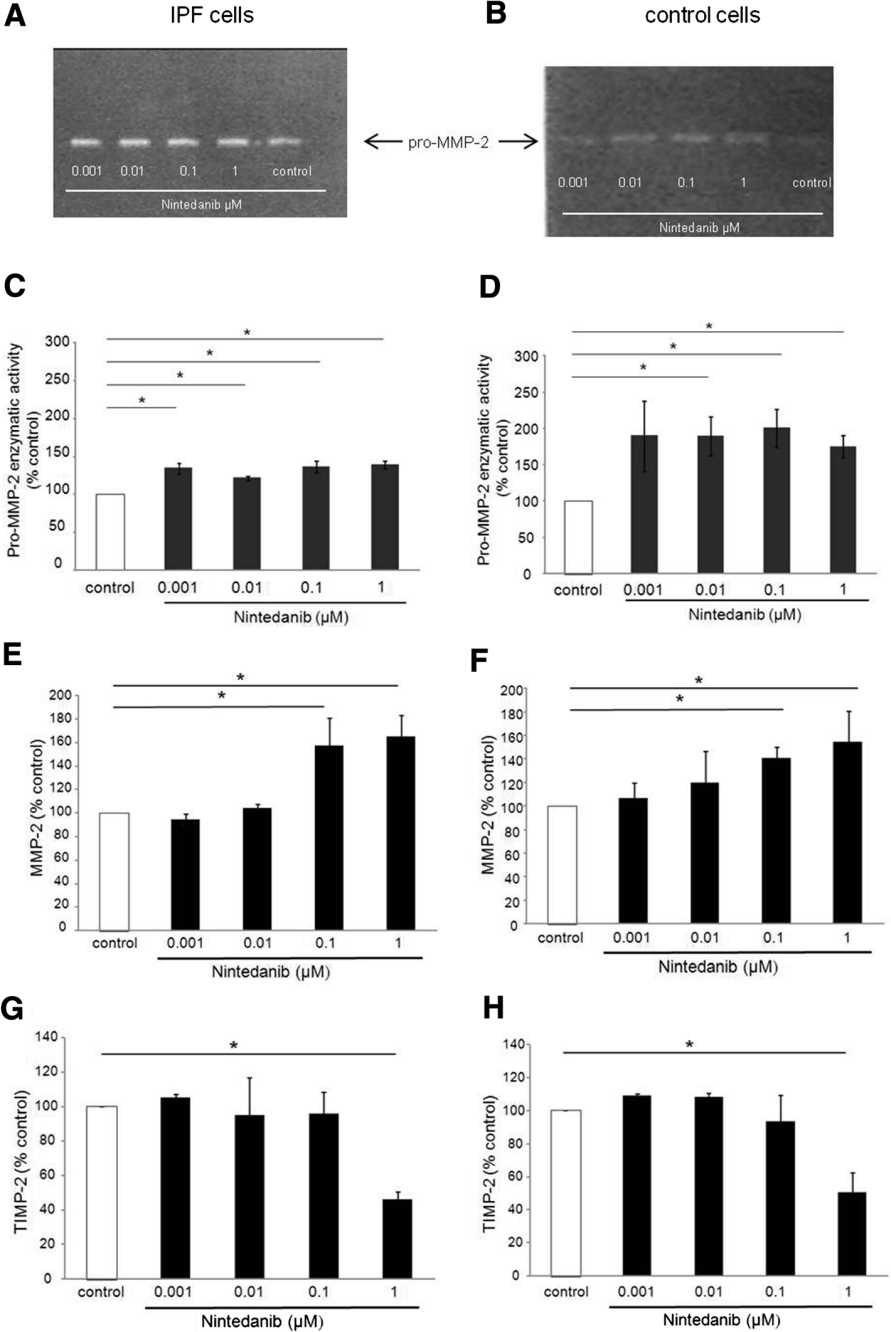
**Effect of nintedanib on enzymatic activity (panel A-D) and secretion of matrix metalloproteinase (MMP)-2 (panel E, F) and tissue inhibitor of metalloproteinase (TIMP)-2 (panel G, H) by primary human lung IPF fibroblasts (A, C, E, G) and by non-fibrotic control cells (B, D, F, H).** Fibroblasts were treated with increasing concentrations of nintedanib (0.001, 0.01, 0.1, and 1 μM) for 24 hours and conditioned cell medium was collected. **(A, B)** Representative gelatine-based zymograms of the effect of nintedanib on pro-MMP-2 enzymatic activity by IPF fibroblasts **(A)** and by control cells **(B)**. Arrows indicate the migration position of purified pro-MMP-2. **(C, D)** Densitometric analysis of pro-MMP-2 enzymatic activity in primary human lung fibroblasts obtained from IPF lungs **(C)**, and from non-fibrotic controls **(D)**. Values are presented as mean ± SEM of independent experiments performed in 4 different cell lines, expressed as percentage of control (serum-free RPMI medium). Each experiment was performed at least in duplicates. *p < 0.05. The effect of nintedanib on the secretion of MMP-2 and TIMP-2 by IPF fibroblasts **(E, G)** and control cells **(F, H)** was assessed by ELISA. Values are presented as mean ± SEM of independent experiments performed in 4 different cell lines, expressed as percentage of control (serum-free RPMI medium). Each experiment was performed at least in duplicates. *p < 0.05.

In IPF fibroblasts the activity of pro-MMP-2 was significantly induced by nintedanib (0.001, 0.01, 0.1, 1 μM), as demonstrated by gelatin zymography (p < 0.05; Figure 3A and C). Similarly, in non-fibrotic control cells nintedanib caused a significant increase of pro-MMP-2 activity (p < 0.05; Figure 3B and D). Only minor amounts of pro-MMP-9 were detected (data not shown). Gelatin zymography data for pro-MMP-2 were confirmed by ELISA (p < 0.05; Figure 3E and F). Finally, IPF fibroblasts, as well as non-fibrotic control cells secreted TIMP-2 protein, which was reduced by nintedanib (1 μM) (Figure 3G,H).

### Growth-factor-induced collagen secretion is inhibited by nintedanib

**Figure 4:**
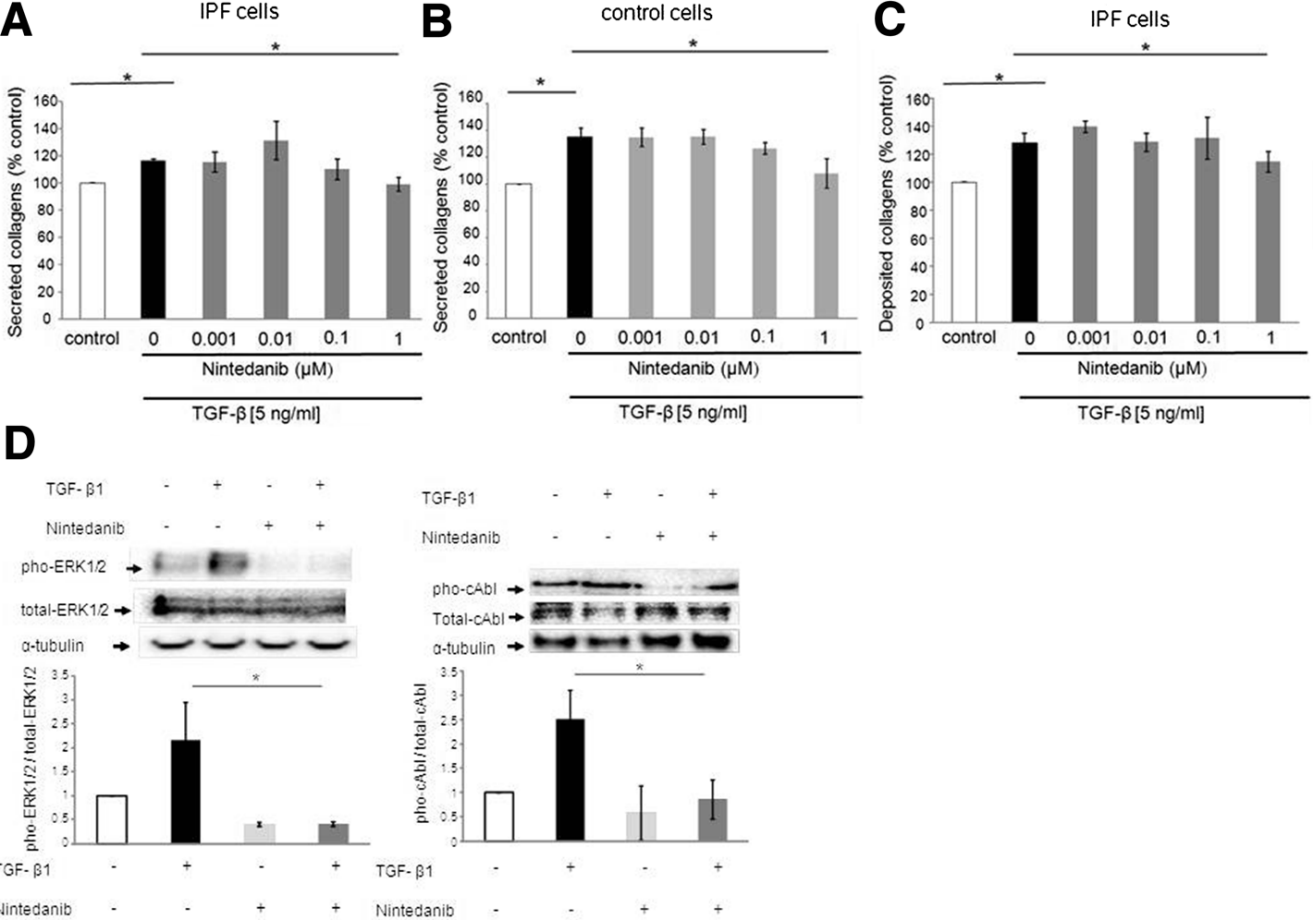
**Effect of nintedanib on TGF-β-induced secretion (panel A, B) and deposition (panel C) of collagens by primary human lung IPF fibroblasts (panel A, C) and by non-fibrotic control cells (B).** Primary human lung fibroblasts were pre-incubated for 30 minutes with nintedanib (0.001, 0.01, 0.1, and 1 μM), before adding TGF-β (5 ng/ml) for 48 hours. Collagen secretion and deposition were quantitated by the Sircol™ Assay, and values are presented as mean ± SEM of independent experiments performed in 4 different cell lines, expressed as percentage of control (0.1% FCS) which was set to 100%. Each experiment was performed at least in duplicates. *p < 0.05. **(D)** Representative immuno-blots showing the expression of total and phosphorylated ERK1/2 and c-Abl in primary human lung IPF fibroblasts. Fibroblasts were starved for 24 hours. Before stimulation with TGF-β1 (5 ng/ml) for 5 minutes, cells were pre-incubated with nintedanib (1 μM) for 30 minutes. The bar charts summarise the densitometric analysis of receptor phosphorylation normalised to α–tubulin and total receptor expression in IPF cells. Data are presented as mean ± SEM of experiment performed in three different cell lines. *p < 0.05.

None of the three growth factors significantly enhanced collagen secretion of primary human lung fibroblasts (data not shown). In contrast, TGF-β1 (5 ng/ml) significantly increased collagen secretion in IPF fibroblasts (p < 0.05; Figure 4A) and in non-fibrotic control cells (p < 0.05; Figure 4B). High concentrations of nintedanib (1 μM) significantly prevented TGF-β-induced collagen secretion from IPF fibroblasts (p < 0.05; Figure 4A), as well as in control cells (p ≤ 0.05; Figure 4B). Equally, in IPF fibroblasts the deposition of collagens was significantly induced by TGF- β1 (5 ng/ml), and this was prevented by 1 μM of nintedanib (p < 0.05; Figure 4C). In control cells, the TGF-β-induced collagen deposition was very low and no significant effect of nintedanib was observed (data not shown). Protein analysis demonstrated an inhibitory effect of nintedanib (1 μM) on TGF-β1-induced phosphorylation of the mitogen-activated protein (MAP) kinase ERK1/2 and the protein tyrosine kinase c-Abl (Figure 4D). No phosphorylation of Smad2/3 was observed upon TGF-β stimulation (data not shown).

## Discussion

In this study we investigated the in vitro effect of nintedanib on the proliferative capacity as well as on the extracellular matrix metabolism of primary human lung fibroblasts obtained from patients with IPF and from non-fibrotic control lungs. We found that the receptor tyrosine kinase inhibitor nintedanib prevented growth-factor induced proliferation of both IPF and control fibroblasts. Furthermore, growth-factor induced collagen synthesis was significantly reduced by nintedanib. Interestingly, the drug up-regulated pro-MMP-2 secretion, but down-regulated its inhibitor TIMP-2.

IPF is a progressive lung disease with a median survival time after diagnosis of 2.5 to 3.5 years [13], and limited therapeutic options. The pathomechanisms that lead to IPF are not fully understood, however, they ultimately result in enhanced numbers of mesenchymal cells and the accumulation of ECM components in the interstitium [1]. Nintedanib, an intracellular inhibitor of tyrosine kinases, has proven good antitumor/antiproliferative efficacy [7], and had beneficial effects in patients with IPF [8]. Nintedanib’s targets include PDGFR, FGFR, and VEGFR [7], which have been shown to be involved in lung fibrosis [4,6,14].

Using primary human lung fibroblasts obtained from IPF lungs and from non-fibrotic control lungs, we found that IPF cells expressed higher levels of PDGFR and FGFR compared to controls. This finding is in line with previous data showing an enhanced expression of FGFR1 on myofibroblast-like cells in IPF patients [5], and up-regulated PDGFRα expression in a rodent model of lung fibrosis [15]. Whereas we found similar levels of VEGFR in fibrotic and non-fibrotic cells, others reported an increased expression of VEGFR1 in IPF patients [16]. However, in the latter study soluble VEGFR was measured in bronchoalveolar lavage fluid, whereas we used protein extracts from pure primary human lung fibroblasts, which makes it difficult to compare the data.

PDGF-BB and bFGF are potent mitogenic and chemotactic factors for mesenchymal cells [2,5] and both factors were shown to be elevated in IPF patients [5,17]. Accordingly, we observed a strong mitogenic effect of PDGF-BB and bFGF in primary human lung fibroblasts from IPF and non-fibrotic control lungs. Importantly, the observed mitogenic effect was stronger in the control cells than in IPF cells (2-fold for PDGF-BB, 1.7-fold for bFGF), which is in line with previous data demonstrating a different response to growth factors by normal and IPF fibroblasts [18]. VEGF is an important regulator of neovascularization [19], and recent data supported the hypothesis that it might be involved in fibrotic diseases [20]. In this regard, marked vascular changes were reported in an animal model of lung fibrosis [21]. Currently, the role of VEGF in IPF is not clear, since VEGF plasma levels were significantly related to radiologic fibrosis scores in patients with idiopathic interstitial pneumonias [20], but decreased VEGF levels in broncho-alveolar lavage fluid from patients with IPF have been reported [22]. Even though VEGF is considered to be a specific mitogen for endothelial cells, we observed a mitogenic effect of VEGF in IPF, as well as in non-fibrotic control fibroblasts, reaching statistical significance only in the latter ones.

In accordance with previous data [7], nintedanib inhibited the PDGF-, FGF-, and VEGF-induced fibroblast proliferation in a concentration dependent manner in IPF as well as in non-fibrotic control cells. In our primary human fibroblasts, we observed that the antagonistic capacity of nintedanib was significantly stronger in non-fibrotic cells compared to IPF fibroblasts. This reduced sensitivity of IPF cells towards nintedanib might be explained by the higher PDGFR and FGFR expression in IPF cells. Furthermore, in contrast to our findings on collagen secretion, where only high concentrations of nintedanib prevented growth-factor induced collagen secretion, cell proliferation was potently inhibited by low concentrations of nintedanib.

The excessive accumulation of ECM is a hallmark of IPF [1], and therefore we studied the effect of nintedanib on ECM metabolism. We found that nintedanib induced the activity and protein levels of MMP-2, and reduced the levels of TIMP-2. MMPs are a family of secreted zinc-containing endopeptidases that degrade proteins of the ECM, and TIMP are their main physiological inhibitors [23]. Both MMP-2 and MMP-9 metabolize various ECM proteins including type IV and type V collagens and gelatin [23]. MMPs are secreted as inactive precursors which have to be activated in the extracellular space. MMP-2 can be activated by type I collagen and thrombin [24,25], but also by a membrane-type MMP-dependent pathway involving TIMP-2 [11]. An imbalance between MMP and TIMP might be involved in the accumulation of ECM in fibrogenesis [26]. Accordingly, a greater increase in the levels of TIMP than levels of MMP-2 was reported and such an imbalance would favour the enhanced deposition of ECM proteins [27]. Interestingly, TGF-β induced lung fibrosis in mice was primarily due to TIMP up-regulation [28], and a recent study demonstrated that over-expression of MMP-9 by alveolar macrophages in mice attenuated the fibrotic reaction after bleomycin instillation [29]. Therefore, if TIMP are elevated in IPF, a drug that reduces the secretion of TIMP-2 by fibroblasts is possibly a powerful tool in the therapy of this devastating disease.

To further investigate nintedanib’s anti-fibrotic potential, we determined the drug’s effect on PDGF-, FGF-, and VEGF-induced collagen secretion. Similar to others we observed no significant modulation of collagen secretion upon stimulation with the three growth-factors in fibroblasts [30,31].

TGF-β is a well-known stimulator of collagen production, a pivotal mediator of fibrogenesis, and TGF-β-induced disturbances are critical in IPF [32,33]. As expected, TGF-β up-regulated total collagens in IPF and control fibroblasts, and this effect was antagonized by the highest concentration of nintedanib (1 μM). To elucidate the underlying mechanism of this effect, several down-stream signalling pathways of TGF-β were studied. It could be demonstrated that nintedanib inhibited TGF-β1-induced phosphorylation of the MAP kinase ERK1/2 and of the protein tyrosine kinase c-Abl. The lack of phosphorylation of Smad2/3 upon TGF-β stimulation in primary human lung fibroblasts is in accordance with previous data [34]. The finding that TGF-β stimulates c-Abl tyrosine kinase is in line with others identifying c-Abl as an important element of TGF-β signalling and crucial for TGF-β-induced synthesis of ECM-proteins [35]. ERK1/2 is a well known component of the non-canonical TGF-β-signalling [36] and inhibition of ERK suppressed collagen expression in vitro [37]. Based on our data we therefore suggest that the antagonistic effect of nintedanib on TGF-β-induced collagen secretion is – at least partly – due to the inhibition of c-Abl and/or ERK1/2. We hypothesize that the kinase inhibitor nintedanib inhibits c-Abl tyrosine kinase, similar to imatinib mesylate, a tyrosine kinase inhibitor specific for PDGFR as well as cAbl [35,38]. Finally, the ERK1/2 MAP kinase is not TGF-β-specific but is also a downstream target responsive to PDGF receptor activation [39]. In summary, our data indicate that nintedanib is not only able to inhibit the pro-fibrotic effects of PDGF and FGF but is also capable of abrogating the effects of TGF-β – a key mediator in tissue fibrogenesis.

Our study has several limitations: i) we are aware that alveolar epithelial cells are thought to be critical in the initiation and progression of the fibrotic process in IPF [1]. However, the growth factor-induced proliferation of local fibroblasts and the exaggerated accumulation of fibroblast-derived ECM ultimately result in the destruction of the lung parenchyma. Therefore, we feel that studying the effect of nintedanib on fibroblasts is justified; ii) we acknowledge that measuring pro-MMPs is not reflecting their bioactivity which requires the interaction with other cell types.

## Conclusions

To the best of our knowledge this is the first study to investigate the effect of nintedanib in primary human lung cells obtained from IPF patients. Our in vitro data demonstrate a significant anti-fibrotic effect of nintedanib in primary human lung fibroblasts derived from patients with IPF. This effect consists of the drug’s anti-proliferative effect, as well as its effect on the ECM, the degradation of which seems to be enhanced. The combined anti-fibrotic activities of nintedanib may impact the progressive course of fibrotic lung diseases like IPF.
